# A biomimetic tumor tissue phantom for validating diffusion‐weighted MRI measurements

**DOI:** 10.1002/mrm.27016

**Published:** 2017-11-20

**Authors:** Damien J. McHugh, Feng‐Lei Zhou, Ian Wimpenny, Gowsihan Poologasundarampillai, Josephine H. Naish, Penny L. Hubbard Cristinacce, Geoffrey J. M. Parker

**Affiliations:** ^1^ Division of Informatics, Imaging and Data Sciences The University of Manchester Manchester UK; ^2^ CRUK and EPSRC Cancer Imaging Centre in Cambridge and Manchester Cambridge and Manchester UK; ^3^ The School of Materials The University of Manchester Manchester UK; ^4^ Research Complex at Harwell Didcot UK; ^5^ Bioxydyn Ltd. Manchester UK

**Keywords:** tumor microstructure, diffusion MRI, biomimetic phantoms, hollow microspheres, coaxial electrospraying

## Abstract

**Purpose:**

To develop a biomimetic tumor tissue phantom which more closely reflects water diffusion in biological tissue than previously used phantoms, and to evaluate the stability of the phantom and its potential as a tool for validating diffusion‐weighted (DW) MRI measurements.

**Methods:**

Coaxial‐electrospraying was used to generate micron‐sized hollow polymer spheres, which mimic cells. The bulk structure was immersed in water, providing a DW‐MRI phantom whose apparent diffusion coefficient (ADC) and microstructural properties were evaluated over a period of 10 months. Independent characterization of the phantom's microstructure was performed using scanning electron microscopy (SEM). The repeatability of the construction process was investigated by generating a second phantom, which underwent high resolution synchrotron‐CT as well as SEM and MR scans.

**Results:**

ADC values were stable (coefficients of variation (CoVs) < 5%), and varied with diffusion time, with average values of 1.44 ± 0.03 µm^2^/ms (Δ = 12 ms) and 1.20 ± 0.05 µm^2^/ms (Δ = 45 ms). Microstructural parameters showed greater variability (CoVs up to 13%), with evidence of bias in sphere size estimates. Similar trends were observed in the second phantom.

**Conclusion:**

A novel biomimetic phantom has been developed and shown to be stable over 10 months. It is envisaged that such phantoms will be used for further investigation of microstructural models relevant to characterizing tumor tissue, and may also find application in evaluating acquisition protocols and comparing DW‐MRI‐derived biomarkers obtained from different scanners at different sites. Magn Reson Med 80:147–158, 2018. © 2017 The Authors Magnetic Resonance in Medicine published by Wiley Periodicals, Inc. on behalf of International Society for Magnetic Resonance in Medicine. This is an open access article under the terms of the Creative Commons Attribution License, which permits use, distribution and reproduction in any medium, provided the original work is properly cited.

## INTRODUCTION

The use of diffusion‐weighted (DW) MRI in oncology is motivated by the potential for inferring clinically useful information related to microstructural properties of tumors from the measured DW signal. Such information typically comes in the form of a biomarker, which may be used for a variety of applications including lesion detection, distinguishing between benign and malignant tissue, and predicting or evaluating response to treatment 1, 2.

Depending on the tissue being imaged and the sequence parameters used for acquisition, a range of biomarkers can be derived from DW data, by modeling the signal in different ways. These models can broadly be split into two categories: phenomenological and biophysical 3. Examples of biomarkers from phenomenological models include the apparent diffusion coefficient (ADC) 4, 5, 6, 7, 8, 9, 10, diffusional kurtosis 11, 12, 13, 14, 15, 16, and the stretched exponential 17, 18. Biophysical models attempt to describe the DW signal in terms of specific microstructural tissue properties, potentially yielding biomarkers such as cell size, intracellular volume fraction, and compartment diffusivities. The greater specificity offered by biophysical models in comparison to phenomenological models has motivated extensive research for white matter applications 19, 20, 21, 22, and recent applications to tumor tissue 23, 24, 25, 26.

If such biomarkers are to become useful clinical tools, it is important that they are subjected to a process of validation to assess both their technical performance, for example their accuracy and precision, and their relationship to biological processes 27. To date, much of the validation of DW‐MRI methods in oncology has focused on the technical validation of ADC using free‐diffusion phantoms. For example, ice‐water phantoms have been used to evaluate the repeatability and reproducibility of ADC values on clinical 28, 29 and preclinical 30 scanners, as well as to investigate spatial variations in ADC due to gradient non‐linearities 31. ADC stability has also been assessed using gels developed with a range of diffusivities and relaxation times 32.

While useful for ADC investigations, free‐diffusion phantoms are not suitable for the validation of other DW‐MRI‐derived biomarkers, as they lack the cellular‐level structure which underlies quantities such as diffusional kurtosis and microstructural parameters. As part of the validation of such biomarkers, it is therefore desirable to have physical phantoms which mimic the cellular structure of tissue, and whose microstructural properties can be controlled and/or characterized. A number of such phantoms have been used for studying diffusion in white matter, including solid fibers 33, hollow silica microcapillaries 34, 35, plant tissue 36, 37, and electrospun hollow fibers 38, but there is a notable absence of systems applicable to tumor tissue 1.

In addition, free‐diffusion phantoms do not capture potentially important ways in which ADC can vary with sequence parameters. For example, free‐diffusion phantoms do not exhibit a dependence of ADC on diffusion time, which is a general phenomenon in biological tissue 39 and has been observed in tumor tissue 40, 41. As such, although ice‐water ADC has been shown to be reproducible across acquisitions with different scan parameters 30, this will not necessarily be the case for tumor ADC.

These considerations motivate the current work, which describes the construction and characterization of a phantom designed as a simple mimic of tumor cellular structure, building on a preliminary report of an earlier phantom design 42. Results from DW‐MRI experiments performed to investigate the phantom's temporal stability are presented, allowing assessment of its potential use as a long‐term test object in multi‐center studies. Experiments designed to evaluate its potential as a tool for validating microstructural measurements and comparing acquisition protocols are also presented, with DW‐MRI characterization compared with independent microstructural measurements 43.

## METHODS

### Phantom Construction and Characterization

**Figure 1:**
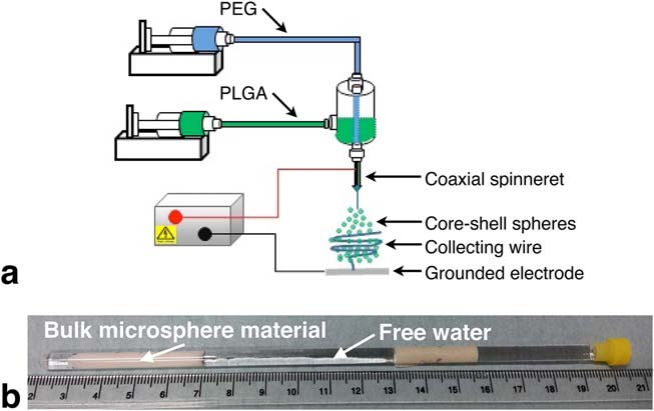
Phantom construction. (**a**) Schematic of the apparatus used to generate the microspheres. Core (polyethylene glycol [PEG]) and shell (poly(d,l‐lactic‐co‐glycolic acid) [PLGA]) solutions were injected into the inner and outer needles of a coaxial spinneret, with resulting hollow spheres collected on a copper wire. (**b**) Bulk phantom used for MR experiments, immersed in water in a 5 mm diameter NMR tube, shown with a centimeter scale.

The phantom consists of a collection of approximately spherical, micron‐scale hollow polymer spheres, which mimic cells. The spheres were produced by coaxial electrospraying 44, extending the approach described previously for generating solid spheres 45; a related technique, coaxial electrospinning, has been used to generate hollow fibers for mimicking white matter 38. Coaxial electrospraying was performed using polyethylene glycol (PEG) and poly(d,l‐lactic‐co‐glycolic acid) (PLGA) for the core and shell of the microspheres, respectively. PEG and PLGA solutions were injected into the inner and outer needles of a coaxial spinneret, at flow rates of 1 and 3 ml/h, respectively. A thin aluminium plate placed 20 cm below the spinneret was used as a ground electrode, and a 12 kV voltage was applied. As the polymer jet travels from the spinneret toward the electrode, the hollow spheres form as the PLGA outer shell rapidly solidifies, with the core solution subsequently evaporating through the shell; this mechanism has been discussed in more detail elsewhere, in the context of spheres generated with a polycaprolactone shell 46. The hollow spheres were collected on a copper wire connected to the ground electrode, generating a bulk sample in approximately 1 h (Fig. 1a). The wire was then removed, leaving the bulk phantom structured as a hollow cylinder approximately 4 cm long, with inner and outer diameters of approximately 1.8 mm and 3 mm.

The phantom was then split into two sections, with one used for MR experiments and the other for characterization with scanning electron microscopy (SEM). The MR sample was placed in a 5 mm NMR tube which was then filled with deionized water (Fig. 1b). At the same time, the SEM sample was also immersed in deionized water in a separate NMR tube. In order to assess the effect of prolonged immersion on the phantom's microstructure, sections of the SEM sample were scanned over a 6‐month period (see https://onlinelibrary.wiley.com/action/downloadSupplement?doi=10.1002%2Fmrm.27016&attachmentId=203701845), quantifying the outer radius of the spheres, *R_o_* 42. The term ‘outer radius’ is used because the SEM measurements reflect the exterior size of the spheres, and do not quantify the non‐zero wall thickness.

The repeatability of the construction process was investigated by generating a second phantom, keeping all electrospraying parameters the same. Similar room temperature and relative humidity conditions were used in both cases (22.6°C, 30% and 23.5°C, 28%), as these variables are known to influence the properties of electrosprayed fibers and particles 47. Analogous to the methods described above for the first phantom, sections of the second phantom were used for MR experiments and SEM analysis. In addition, high resolution synchrotron‐CT (sCT) scans were performed on two sections of the second phantom, with one section immersed in water and one kept dry; full details of the sCT acquisition and analysis are given in the Supporting Information. Briefly, these scans were used to characterize the sphere wall thickness (from manual measurements, https://onlinelibrary.wiley.com/action/downloadSupplement?doi=10.1002%2Fmrm.27016&attachmentId=203701845) and the sphere volume fraction (from segmenting the images into ‘sphere’ and ‘non‐sphere’ regions, https://onlinelibrary.wiley.com/action/downloadSupplement?doi=10.1002%2Fmrm.27016&attachmentId=203701845). The first and second phantoms will be referred to as phantoms A and B, respectively.

### MR Acquisition

MR experiments with phantom A were carried out over a period of approximately 10 months, at the following post‐immersion time points: ∼2, 6, 24, 72 h, 1, 2, 3, 4, 9, 16, 20, 26, and 42 weeks. All time points except the first and last had corresponding SEM analysis. Scans with phantom B were carried out over 1 month, at ∼7, 27 h, 1 and 4 weeks post‐immersion. All scans were performed on a 7 T horizontal bore magnet (Magnex Scientific Ltd., Abingdon, UK) interfaced to a Bruker Avance III console (Bruker BioSpin, Ettlingen, Germany), with the phantom(s) and a control NMR tube containing only deionized water placed inside a transmit/receive volume coil. Each scan session included pulsed gradient spin‐echo (PGSE) acquisitions for evaluating ADC and microstructural parameters. Room temperature was monitored and varied by a maximum of 0.7°C within a given scan session, with a mean ± standard deviation (SD) of 24 ± 1°C over all time points.

For ADC calculations, DW data were acquired with *b* = 0, 150, 500, 1000 s/mm^2^, *δ* = 4 ms, Δ = 12 ms (*G* = 0, 117.6, 214.7, 303.7 mT/m) and 45 ms (*G* = 0, 58.3, 106.4, 150.5 mT/m), with TE = 21.3, 54.3 ms, respectively, and TR = 2500 ms. In addition, DW data were also acquired with *G* = 0, 70, 140, 210 mT/m, *δ* = 4 ms, Δ = 12, 23, 45 ms, with TE = 21.3, 32.3, 54.3 ms, respectively, and TR = 2500 ms; *b*‐values were 53.1, 212.5, 478.1, 107.5, 430.0, 967.6, 216.3, 865.1, 1946.5 s/mm^2^. In a subset of the experiments, the Δ = 12 ms ADC acquisition was repeated at the end of the scan session, to assess short‐term (∼2 h) repeatability. All imaging data were acquired with a 30 mm × 30 mm field of view, 128 × 128 matrix, and 10 axial slices of 1 mm thickness.

### MR Analysis

ADC maps for Δ = 12 and 45 ms were generated using maximum likelihood (ML) fitting 48, with the noise, σ, estimated from a region of interest (ROI) drawn in the background: $\sigma =S_{\text{bg}}\sqrt{2/\pi }$, where *S*_bg_ is the mean signal intensity in the background ROI 49. Using a single Rician probability density function (PDF) in the objective function was appropriate here as the signals used for ADC fitting were not averaged 50. The phantom material region was obtained using a semi‐automated method which first separates the NMR tubes from the background, then thresholds the *b* = 0 s/mm^2^ images to remove high signal voxels corresponding to free water; minor manual adjustment then provided the phantom ROI. Free water ADC values were obtained from the control NMR tube. The signal‐to‐noise ratio (SNR) was calculated as *S_b_*_0_∕σ, where *S_b_*_0_ is the mean *b* = 0 s/mm^2^ signal in the phantom.

For the multi‐*G*, multi‐Δ dataset, signals for each Δ acquisition were normalized to their *G* = 0 mT/m scan, and these normalized signals were analyzed by fitting a two‐compartment microstructural model combining restricted diffusion inside a sphere with hindered extra‐sphere (analogous to extra‐cellular) diffusion, yielding three model parameters: sphere radius, *R*, intra‐sphere (analogous to intra‐cellular) volume fraction, *f_i_*, and free diffusivity, *D*. As the same fluid is inside and outside of the spheres, a single diffusivity was used in the model. The normalized DW‐MRI signal, *S*∕*S*_0_, is given by

(1) $$S/S_{0}=f_{i}S_{i}+(1-f_{i})S_{e},$$

where

(2) $$S_{i}=\text{exp}(-2\gamma^{2}G^{2}\sum_{m=1}^{\infty }\frac{1}{\alpha_{m}^{2}(\alpha_{m}^{2}R^{2}-2)}[\frac{2\delta }{\alpha_{m}^{2}D}+\frac{2e^{-\alpha_{m}^{2}D\delta }+2e^{-\alpha_{m}^{2}D\Delta }-e^{-\alpha_{m}^{2}D(\Delta -\delta )}-e^{-\alpha_{m}^{2}D(\Delta +\delta )}-2}{\alpha_{m}^{4}D^{2}}]),$$

(3) $$S_{e}=\exp\left(-\gamma^{2}\delta^{2}G^{2}(\Delta -\delta /3)\frac{D}{1+f_{i}/2}\right).$$

Equation (2) is the PGSE signal for diffusion restricted within an impermeable sphere, assuming a Gaussian phase distribution; *α_m_* is obtained from the *m*th root of $\alpha_{m}RJ\prime_{3/2}(\alpha_{m}R)-\frac{1}{2}J_{3/2}(\alpha_{m}R)=0$, where *J*_3∕2_ is the Bessel function of the first kind, order 3/2 51, 52. Equation (3) gives the signal for hindered extracellular diffusion with the diffusivity reduced by a tortuosity factor, 1 + *f_i_*/2 53.

Two fitting procedures were performed: first, all model parameters were estimated in the fitting; second, *D* was fixed to the median ADC (at Δ = 12 ms) measured in the water‐only NMR tube, which serves as a ground truth for *D*. For both procedures, fitting was performed both on a voxel‐wise basis and using whole‐ROI averaged signals (fitting to the mean signal from the entire phantom ROI). In each case, fitting was performed for 100 starting values, taking the final result as the fit with the lowest value of the objective function. As the microstructural model fitting involves averaging and/or normalizing signals, a single Rician PDF no longer characterizes the distribution of the signals and cannot be used in the objective function, making the ML fitting method described above no longer appropriate. Instead, least squares fitting was used, with potential bias mitigated by discarding signals lower than 2*S*_noise,_ where *S*_noise_ is the mean signal in a noise ROI 54. Fitting was performed using a Nelder‐Mead simplex algorithm, with parameters constrained to be within plausible biological limits: 0.1 ≤ *R* ≤ 25 µm, 0.1 ≤ *D* ≤ 3 µm^2^/ms, 0.01 ≤ *f_i_* ≤ 1. When fitting to whole‐ROI averaged signals, the precision of the model parameters was assessed by bootstrapping the residuals. Due to the SNR differences for different Δ acquisitions, bootstrapped datasets were generated for a given Δ using the residuals for that Δ; for example, a residual for a Δ = 45 ms data point would not be added to a Δ = 12 ms data point. One thousand bootstrap samples were generated, and 95% confidence interval (CI) limits were taken as the 2.5% and 97.5% quantiles of the bootstrap distribution 55. The bootstrapping results were also used to investigate correlations between the model parameters. The effect of acquisition protocol on microstructural estimates was assessed by fitting the model to whole‐ROI averaged signals using only the data from the Δ = 12 ms and 23 ms acquisitions, that is, excluding all data from the longest diffusion time.

Coefficients of variation (CoVs) were calculated to assess measurement repeatability, and two‐sample *t*‐tests were used for statistical analyses, with *P* < 0.05 taken to indicate significant differences. All analyses were carried out with MATLAB 2014a (The MathWorks, Inc., Natick, MA).

## RESULTS

### SEM Characterization of Phantom Microstructure and Stability

**Figure 2:**
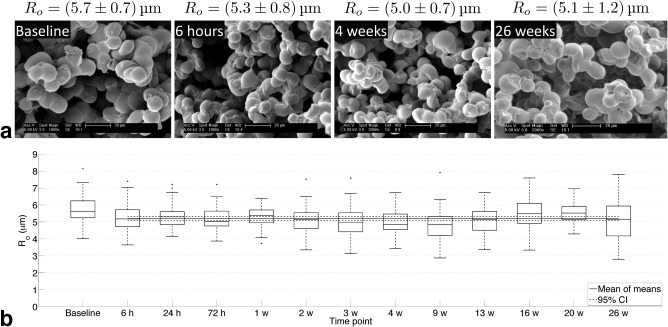
SEM characterization of phantom A microstructure. (**a**) Example images at baseline (before immersion) and three post‐immersion time points, with mean ± SD outer radius values. (**b**) Box plots of outer radii determined from SEM images, for all post‐immersion time points; solid line shows the mean of mean values, and dashed lines show 95% CI. Note that time points are plotted evenly spaced, as opposed to on an absolute time scale.

SEM images (Fig. 2a, phantom A) show that the spheres tend to group together, indicating that the phantom's microstructure is not simply a packing of discrete idealized spheres, but consists of extended clumps of spheres. The mean ± SD of the baseline radii was 5.7 ± 0.7 µm. The CoV of the mean post‐immersion values was 4.2%, and the maximum difference in means between the first post‐immersion time point and subsequent points was 0.46 µm. Averaging over the mean values at each post‐immersion time point gave *R*_o_ = 5.2 ± 0.2 µm, which was taken as the ground truth outer sphere radius for phantom A (Fig. 2b). For phantom B, measurements over 1 month gave a CoV of 4.7% and *R*_o_ = 5.6 ± 0.3 µm. Comparing *R*_o_ values for the two phantoms at equivalent post‐immersion time points gave a mean difference of 0.4 µm.

### Stability and Time‐Dependence of Phantom ADC

**Figure 3:**
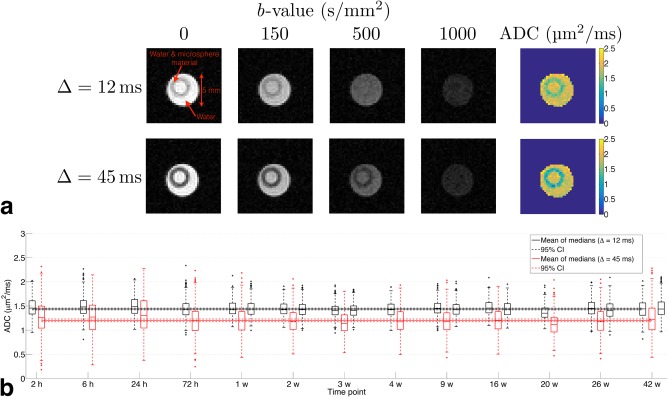
Phantom A ADC. (**a**) Example DW images and ADC maps for the two diffusion times, acquired at 6 h post‐immersion. As the bulk phantom is structured as a hollow cylinder, the phantom appears as an annulus on these axial slices. (**b**) Box plots of ADC at Δ = 12 ms (black) and 45 ms (red) at each time point; solid lines show the mean of median values, and dashed lines show 95% CI. Note that time points are plotted evenly spaced, as opposed to on an absolute time scale. Seven time points have two Δ = 12 ms scans separated by approximately 2 h, to investigate short‐term repeatability.

Figure 3a shows example DW images and ADC maps for phantom A at the 6‐h time point. The lower signal annular region corresponds to the phantom, with the water in the center filling the space left by the wire used to collect the spheres during production. Mean ± SD SNR in phantom A, averaged over all slices and time points, was 27 ± 3 and 17 ± 2 for the first Δ = 12 ms and the Δ = 45 ms scans, respectively. ADC was consistently higher at the shorter diffusion time, with a mean ± SD of ROI median values over each time point of 1.44 ± 0.03 µm^2^/ms and 1.20 ± 0.05 µm^2^/ms for the first Δ = 12 ms and the Δ = 45 ms scans, respectively (Fig. 3b). Median ADC values at the two diffusion times were significantly different (*P* < 0.001), with a mean percentage difference of 16%. Such a dependence was not observed in the free water, where the mean ± SD of ROI median values over each time point was 2.01 ± 0.04 µm^2^/ms and 2.01 ± 0.03 µm^2^/ms for the first Δ = 12 ms and the Δ = 45 ms scans, respectively (*P* = 0.62).

Figure 3b also demonstrates the stability of ADC values over the 10‐month period, with CoVs of 2.4% and 4.3% for Δ = 12 ms and 45 ms, respectively. In the seven scan sessions where the Δ = 12 ms acquisition was repeated, the mean absolute percentage difference in median ADC values in the phantom was 1.1%, and no significant difference was found (*P* = 0.18). While weeks 9, 16, and 26 showed a trend for a lower ADC at the end of the experiment, both in the phantom and free water, this was not observed consistently.

Phantom B also exhibited stable ADC values, with CoVs of 1.0% and 2.1% for Δ = 12 ms and 45 ms, respectively, over a month. ADC values for both diffusion times were lower than in phantom A, with 1.32 ± 0.01 µm^2^/ms and 1.02 ± 0.02 µm^2^/ms for the first Δ = 12 ms and the Δ = 45 ms scans, respectively.

### Application of Phantom for Microstructural Model Evaluation

**Figure 4:**
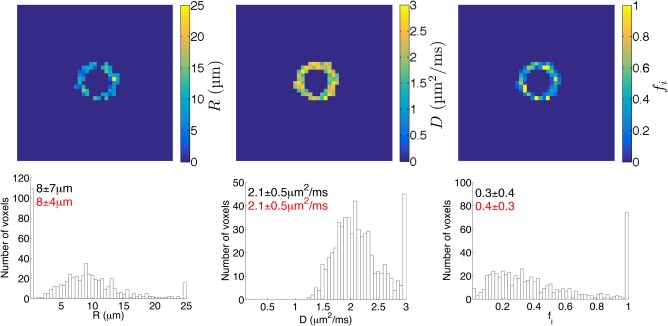
Voxel‐wise microstructural estimates, for phantom A. Top row: example maps of each model parameter, when fitting all parameters; images are a representative slice from the 1‐week time point. Bottom row: histograms of each model parameter, when fitting all parameters. Quoted values are the median ± IQR, for all phantom voxels (black) and, where applicable, excluding voxels where at least one parameter value was within 1% of the fit constraints (red).

Figure 4 shows example maps and histograms of *R*, *D*, and *f_i_*, at the 1‐week time point for phantom A. Large variations in parameter values were observed, indicating that voxel‐wise estimates had poor precision. In particular, fits in a number of voxels resulted in values at or near the fit constraints. For example, at the 1‐week time point the percentage of voxels with values within 1% of the constraints was 21%, 8%, and 14% for *R*, *D*, and *f_i_*, respectively. Fixing *D* had little impact on these percentages (for *R* and *f_i_*), showing that fixing the diffusivity did not improve the precision of parameter estimates (https://onlinelibrary.wiley.com/action/downloadSupplement?doi=10.1002%2Fmrm.27016&attachmentId=203701845). The relatively low spread in *R*_o_ values from SEM suggests that the variation in *R* stems from imprecision, rather than reflecting genuine heterogeneity in the phantom. In general, parameter estimates suffered from poor precision with voxel‐wise fitting, an observation which was consistent across time points.

**Figure 5:**
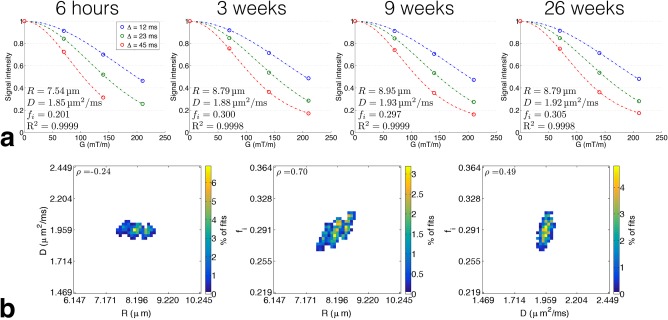
Microstructural estimates from fitting to whole‐ROI averaged signals, for phantom A. (**a**) Example fits (dashed lines) for a range of time points, with model parameters and coefficient of determination, R^2^, shown in each panel. Signals (circles) are plotted as a function of *G* (*x*‐axis) for different Δ (colors). (**b**) Correlations between parameters, obtained from bootstrap simulations and plotted as bivariate histograms. Plots are from the 1‐week time point, and Pearson's correlation coefficient, *ρ*, is quoted in each panel.

Figure 5a shows example fits using whole‐ROI averaged signals, when fitting all parameters; model parameters and the coefficient of determination, R^2^, are shown in each case. The model fits the data well, with a mean R^2^ value (over all time points) of 0.9998 for both fitting procedures. At 6 and 72 h, and 42 weeks, the 2*S*_noise_ threshold applied to remove low SNR data points resulted in the highest‐*G*, highest‐Δ signal being excluded from the fitting, while it was included at all other time points. Compared with fits where this data point was explicitly excluded, including it for weeks 1–20 tended to slightly increase *R* estimates and slightly decrease *D* estimates (mean percentage differences of 5% and −2%, respectively), with negligible effect on *f_i_*; at week 26 it had negligible effect on any parameter. For consistency across time points, subsequent analyses focus on fits where the highest‐*G*, highest‐Δ signal was always excluded. Mean R^2^ values for these fits were 0.9998 (fitting all parameters) and 0.9996 (fixing *D*).

Example parameter correlations, obtained from bootstrapping, are shown in Figure 5b, where the bootstrapped parameter values for the 1‐week time point are plotted as bivariate histograms. Here, the strongest correlation was between *R* and *f_i_* (*ρ* = 0.70, Pearson's correlation coefficient), reflecting the fact that larger cells with higher volume fractions and smaller cells with lower volume fractions can give rise to similar signals. Trends in correlations over time were broadly consistent with those in Figure 5b, except at 6 h and 9 weeks, where positive correlations between *R* and *D* were observed (*ρ* = 0.14, 0.11), along with stronger correlations between *D* and *f_i_* (*ρ* = 0.74, 0.81).

**Figure 6:**
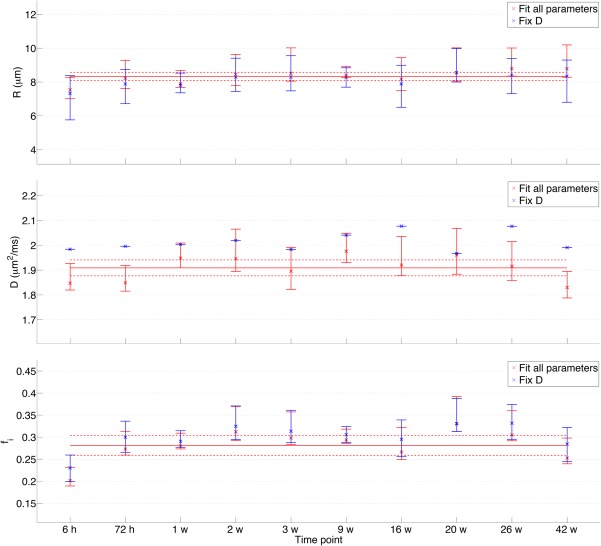
Microstructural estimates for phantom A (excluding the 24‐h and 4‐week time points, due to misalignment between acquisitions and severe image artefacts, respectively). Data points are the values obtained from whole‐ROI averaged fitting, and error bars represent 95% CI limits obtained from bootstrapping. Results from the two fitting procedures are shown in different colors; when *D* is fixed (blue points), *D* values have no error associated with them from bootstrapping. Solid lines show mean parameter estimates over all time points, and dashed lines show 95% CI. Note that time points are plotted evenly spaced, as opposed to on an absolute time scale.

Phantom A's microstructural estimates are shown in Figure 6. *R* was consistently overestimated compared with SEM measurements of *R*_o_. When fitting all parameters, the mean ± SD over all time points for *R* was 8.3 ± 0.4 µm, compared with 5.2 ± 0.2 µm from SEM. Mean ± SD over all time points for *D* was 1.91 ± 0.05 µm^2^/ms, showing that *D* was consistently underestimated compared with the free water ADC of 2.01 ± 0.04 µm^2^/ms (*P* < 0.001, comparing median free water ADCs from the first Δ = 12 ms acquisition and *D* values from whole‐ROI averaged fitting). Fixing *D* resulted in slightly lower *R* and higher *f_i_* estimates, trends expected based on the correlations shown in Figure 5b. Note that as *D* was fixed to the free water ADC measured at each time point, the blue data points in Figure 6's central plot reflect the variation in free water ADC (CoV = 1.8%).

**Figure 7:**
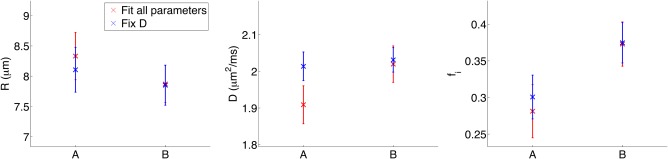
Microstructural estimates for phantoms A and B, for both fitting procedures. Mean ± SD values are plotted, averaged over 10 months and 1 month for A and B, respectively.

For phantom B, *R* = 7.9 ± 0.3 µm, again overestimated compared with *R*_o_ = 5.6 ± 0.3 µm from SEM. In contrast to phantom A, *D* for phantom B was consistent with free water ADC; fixing *D* therefore had less impact on *R* and *f_i_* than for phantom A. Figure 7 plots microstructural estimates for both phantoms, averaged over 10 months (A) and 1 month (B), for both fitting procedures. In terms of percentage differences, the intracellular volume fraction differs most between the two phantoms, with *f_i_* values significantly higher for B (*P* < 0.01 for both fitting methods).

For both fitting procedures, *f_i_* showed the greatest variability, with the other parameters yielding CoVs of less than 5% (Table 1). Fixing *D* had the greatest effect on the *f_i_* CoV for phantom A, with a reduction by a factor of 1.3 compared with fitting all parameters, but in general fixing *D* had little impact on the stability of *R* or *f_i_*.

**Table 1:** CoVs for microstructural parameters from phantoms A and B, for both fitting procedures.

	*R* CoV (%)		*D* CoV (%)		*f_*i*_* CoV (%)	
	A	B	A	B	A	B
Fit all parameters	4.7	3.9	2.7	2.5	13	8.1
Fix *D*	4.6	4.2	1.9	1.6	9.9	7.3

Repeating the fitting with whole‐ROI averaged signals from only the Δ = 12 ms and 23 ms acquisitions resulted in lower mean *R* and *f_i_* values for both phantoms when fitting all parameters (decreases ∼ 15–20% for *R* and *f_i_* relative to fitting to the full dataset).

**Figure 8:**
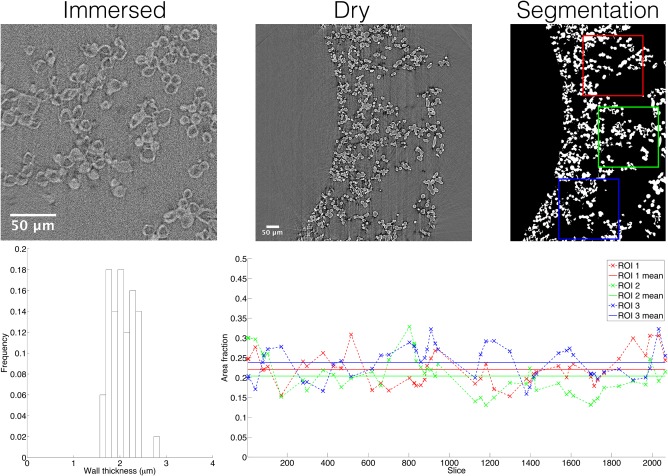
sCT images of phantom B. Example images are shown for immersed and dry spheres, along with an example segmentation of a dry sphere image. The wall thickness histogram (from measurements of 50 spheres) and area fraction estimates (from measurements on 50 slices, with 3 ROIs per slice) are shown on the bottom row.

sCT images obtained from sections of phantom B showed a clear difference in contrast between the immersed and dry conditions, with the hollow structure of the spheres evident in the immersed state (Fig. 8). This difference in contrast is hypothesized to be related to the influence of water on the core polymer, PEG, which is water‐soluble, and as such is expected to dissolve when the spheres are immersed. The wall thickness, estimated from manual measurements of 50 spheres in the immersed state (https://onlinelibrary.wiley.com/action/downloadSupplement?doi=10.1002%2Fmrm.27016&attachmentId=203701845), was 2.1 ± 0.3 µm. Note that such measurements will tend to overestimate the true wall thickness, as the slices cut through spheres at different angles 56. Although the manual method used for the thickness measurements does not satisfy all criteria for using stereological corrections, applying the *π*/4 factor stated in 56 brings the estimate down to 1.6 µm, which is consistent with the lower end of the measured values (see histogram in Fig. 8), and is likely more representative of the wall thickness. Figure 8 also shows an example of the segmentation obtained from one slice of the dry spheres (also see https://onlinelibrary.wiley.com/action/downloadSupplement?doi=10.1002%2Fmrm.27016&attachmentId=203701845). From area fraction measurements from 150 ROIs, the sphere volume fraction was estimated as 0.22 ± 0.05 (Fig. 8).

## DISCUSSION

The low CoV of mean *R*_o_ values in phantom A, and the fact that the maximum difference post‐immersion was less than half a micron, suggest that the phantom microstructure shows little variation over 6 months. Although phantom A's baseline *R*_o_ was higher than all post‐immersion time points, a trend not observed in phantom B, the similarity in post‐immersion *R*_o_ values for the two phantoms provides evidence of the repeatability of the construction process, in that samples with comparable sphere sizes can be generated. SEM characterization demonstrates the stability of both phantoms, and also shows that the sphere size is appropriate for mimicking tumor cells 25, 57.

The observed tendency for the spheres to group together has both negative and positive implications. On the one hand, the aggregation is not ideal in that such a structure is not generally considered in the types of microstructural model the phantom is designed to validate, where tissue is modeled as a collection of individual spherical cells. But on the other hand, the aggregation may better reflect biological tissue, where cell adhesion plays an important role in forming and maintaining tissue structure 58. It is hypothesized that the extent of the aggregation in the bulk phantom depends on the electrospraying parameters, and further work is required to assess the degree to which it can be controlled. While the size and aggregation of the spheres show similarity to tumor tissue, it should be noted that the current phantom clearly oversimplifies the tumor microenvironment. For example, structures such as cell nuclei, collagen fibers, and blood vessels are not mimicked, nor are different cell populations, such as tumor and immune cells. While future work could increase the complexity of the phantom, the current phantom still has utility in investigating microstructural models which are themselves simplifications and do not attempt to capture all aspects of tumor microstructure. By controlling the phantom's ground truth microstructure and investigating the resulting DW‐MRI parameters, insight may be gained into the sensitivity of DW‐MRI to different aspects of tissue structure, with the phantoms potentially aiding the development of microstructural models.

The observation of a higher ADC at a lower diffusion time provides evidence of hindered and/or restricted diffusion in the phantom, consistent with the dependence of ADC on diffusion time that has previously been observed in biological tissue in general and in tumor tissue specifically. Also, the absence of such a dependence in freely diffusing water is expected due to the lack of structures to impede diffusion, as is the case with the free‐diffusion phantoms previously used for DW‐MRI validation (e.g., ice‐water and gels). These findings, along with the observation that the multi‐*G*, multi‐Δ data are well described by a microstructural model, demonstrate that this phantom more closely reflects diffusion in tissue than previous phantoms, and emphasize the need to consider diffusion times, and not only *b*‐values, when comparing ADC values across studies. For example, if different studies use different diffusion times but the same *b*‐values, in vivo ADC values may differ while free‐diffusion phantoms may give comparable values. As such, it should be emphasized that *b*‐values alone do not fully characterize a scan when measuring diffusion in systems such as biological tissue 59, and this should be considered when discussing standardizing acquisitions 1, 2. Using the biomimetic phantom in multi‐center studies could therefore enable a more comprehensive validation of DW‐MRI, enabling comparison of ADC time‐dependence, and microstructural estimates, between scanners. This would be another way of addressing the recently noted need for ‘more data on interplatform reproducibility’ 2, in addition to studies utilizing free‐diffusion phantoms and healthy volunteers 60, 61. The fact that this is a water‐based phantom is advantageous for multi‐center studies, as the phantoms could be distributed dry and immersed in water on‐site. This is more practical than using phantoms immersed in organic solvents 38, 42, which require the use of protective clothing and fume cupboards. Although the structural robustness of the phantoms has not been fully evaluated, initial experience suggests that they can be successfully transported between sites without obvious degradation. The small size of the bulk phantom is a limitation in terms of its use on clinical scanners, making the current phantom better suited to preclinical scanners. Developing larger phantoms suitable for clinical scanners is a focus of ongoing work.

The CoVs of less than 5% indicate that ADC repeatability in the phantom is very good, and provide evidence that the phantom remains stable over 10 months. As PLGA is known to degrade over time, with many factors influencing the degradation process 62, 63, it is expected that the phantom will eventually become unstable, potentially limiting the extent to which it can be used as a long‐term test object. This study suggests that the phantom can be used for at least 10 months, with further longitudinal analysis required to track longer‐term stability.

While SEM characterization showed that phantoms A and B had comparable sphere sizes, providing evidence of the repeatability of the construction process, the ADC differences suggest that the underlying microstructure does vary between the two phantoms. The microstructural modelling suggests that phantom B has a higher *f_i_* than phantom A, which is consistent with the observed lower ADC in phantom B, as ADC is expected to decrease as intracellular volume fraction increases 64, 65. This suggests that phantom A's sphere volume fraction is lower than 0.22, the sCT‐derived volume fraction for phantom B, although the lack of a ground truth volume fraction for phantom A precludes a full validation of this finding. Note that as the sphere volume fraction obtained from the segmented sCT images includes both the sphere wall and the hollow interior, it is expected to be larger than the MR‐derived *f_i_*, which is taken to reflect only the hollow interior. As such, there is a clear overestimate from the microstructural model, which yields *f_i_* = 0.37 ± 0.03 for phantom B.

While the similarity of MR‐estimated radii for the two phantoms is consistent with the similarity observed on SEM, the difference in absolute values between the two modalities indicates the microstructural model also overestimates the sphere size. As *R* is expected to reflect the inner radius, as opposed to the outer radius seen with SEM, the overestimation is even greater when considering the relatively thick walls observed with sCT. Microstructural modelling in white matter has revealed a tendency to overestimate axonal radii 21, with recent work suggesting that this trend may be driven by the unmodeled influence of time‐dependent extracellular diffusion 66. However, the time‐dependence of extracellular diffusion is expected to be weaker in three‐dimensional geometries, such as the sphere packings considered in the present work, than in the two‐dimensional geometries relevant to axonal packings 25. As such, neglecting such time‐dependence may have less of an impact on compartment size estimates in three‐dimensions, and the present work's use of a time‐independent extracellular diffusivity is consistent with that used in previous approaches to tumor microstructural modelling 23, 25.

Exchange of water across the sphere wall is another unmodeled effect that may contribute to bias in *R* and *f_i_* 67. As longer diffusion times are expected to increase sensitivity to exchange, it may be hypothesized that, depending on the rate of exchange, excluding the longest diffusion time data from the fitting would reduce such sensitivity and therefore improve the accuracy of *R*. Excluding the Δ = 45 ms data did reduce *R*, bringing the estimate closer to the SEM *R*_o_, though a bias still remains, suggesting that sensitivity to exchange may still be present at Δ = 23 ms. As faster exchange is expected to lead to a greater underestimate in *f_i_* if not accounted for 67, it may be hypothesized that *f_i_* estimates would increase when using an acquisition less sensitive to exchange, such as excluding longer diffusion times; however, the opposite trend was observed here, suggesting that exchange is not the dominant effect on *f_i_* estimates. Moreover, the sCT data suggests that *f_i_* is overestimated, not underestimated, from the microstructural model. Again, further work, such as a filter exchange imaging (FEXI) experiment 68, is needed to characterize the permeability of the spheres in order to assess these effects, and, more generally, the dependence of microstructural estimates on acquisition parameters warrants further investigation. Another factor which may influence the microstructural parameters is the presence of large pores in the extracellular space, due to the extended clumping of the spheres. Depending on the size of these regions in relation to the diffusivity and diffusion time, water here may appear restricted at longer diffusion times, therefore contributing to the proportion of restricted signal, which would increase *f_i_* and may also increase *R*. This effect is also consistent with *f_i_* decreasing when excluding the longest diffusion time, as diffusion in these regions may appear free, again, depending on their size.

Compartmental differences in T_2_ may also contribute to the observed bias in *R* and *f_i_*, with simulations (not shown) indicating that if T_2_ is higher inside than outside the spheres, all model parameters are overestimated, with the magnitude of the bias increasing as the T_2_ difference increases. Such an effect is consistent with the experimentally observed overestimation of *R* and *f_i_*, although an overestimation of *D* was not seen. A combination of compartmental differences in T_2_ and permeability may influence the model parameters, and further work is needed to understand potential surface effects as water interacts with the spheres’ inner and outer shells.

A limitation of the comparisons between modalities is that different sections of the bulk phantom have been used for sCT, SEM and MR, and further work is required to characterize the homogeneity of the microstructure within a given sample as well as between different samples. Such developments would enhance the utility of the phantoms as tools for validating DW‐MRI microstructural measurements.

## CONCLUSIONS

A novel biomimetic tumor tissue phantom has been developed and shown to be stable over a period of 10 months. The phantom exhibits time‐dependent diffusion and signals are well described by a microstructural model, indicating that the phantom more closely reflects diffusion in tissue than previously used free‐diffusion phantoms. Microstructural estimates were found to be more variable than ADC measurements, with evidence of bias in *R* and *f_i_*. It is envisaged that such phantoms will be used for further investigation of microstructural models relevant to characterizing tumor tissue, and may also find application in evaluating acquisition protocols and comparing DW‐MRI‐derived biomarkers obtained from different scanners at different sites.

## Supporting information


**Fig S1.** Wall thickness measurement for phantom B. Four measurements were made for each sphere, with positions guided by perpendicular lines through the sphere centre, as well as by comparing overlays on original (left) and mean filtered (right) images.
**Fig S2.** sCT segmentation for estimating phantom B's sphere volume fraction. On a single cropped slice, manual labelling of ‘sphere’ (green) and ‘non‐sphere’ (red) regions was performed, and used to train a classifier to segment the entire image. The classifier was then applied to 49 other slices, providing segmentations from which regional area fractions, *a_*f*_*, were obtained.
**Fig S3.** Voxel‐wise microstructural estimates, for phantom A. (a) Example maps of each model parameter are shown (columns) for two fitting procedures (rows). Images are a representative slice from the 1 week time point. (b) Histograms of each model parameter (columns) for two fitting procedures (rows). Quoted values are the median ± IQR, for all phantom voxels (black) and, where applicable, excluding voxels where at least one parameter value was within 1% of the fit constraints (red). The first and third row form Figure 4 in the main text.
