# Supplementary material for: A biomimetic tumor tissue phantom for validating diffusion‐weighted MRI measurements
Source: Magn Reson Med. 2017 Nov 20;80(1):147–58. doi: 10.1002/mrm.27016 (PMC5900984; doi:10.1002/mrm.27016)
Supplement: Supplementary file 1 — Fig S1. Wall thickness measurement for phantom B. Four measurements were made for each sphere, with positions guided by perpendicular lines through the sphere centre, as well as by comparing overlays on original (left) and mean filtered (right) images. Fig S2. sCT segmentation for estimating phantom B's sphere volume fraction. On a single cropped slice, manual labelling of ‘sphere’ (green) and ‘non‐sphere’ (red) regions was performed, and used to train a classifier to segment the entire image. The classifier was then applied to 49 other slices, providing segmentations from which regional area fractions, af, were obtained. Fig S3. Voxel‐wise microstructural estimates, for phantom A. (a) Example maps of each model parameter are shown (columns) for two fitting procedures (rows). Images are a representative slice from the 1 week time point. (b) Histograms of each model parameter (columns) for two fitting procedures (rows). Quoted values are the median ± IQR, for all phantom voxels (black) and, where applicable, excluding voxels where at least one parameter value was within 1% of the fit constraints (red). The first and third row form Figure 4 in the main text. [file MRM-80-147-s001.pdf]

Supporting information

SEM preparation

For both phantoms, the SEM sample was immersed in deionised water at the same time as the corresponding MR sample. In order to assess the effect of prolonged immersion on the phantom's microstructure, sections of the SEM sample were scanned over periods of 6 months (phantom A) and 1 month (phantom B).

As materials must be dry in order to undergo SEM, small sections of the phantom were removed and then dried overnight, prior to SEM. Removal of these small sections was timed to coincide with the MR time points, with sections taken out of the water less than 1 hour after the start of the MR scans; the SEM analysis therefore provided a ground truth for the MR measurements at each time point. Note that a new section of the SEM sample was removed at each time point, so that sections undergoing SEM were only dried once as opposed to undergoing repeated cycles of immersion and drying.

Synchrotron-CT acquisition

Sample preparation for scanning by computed tomography

Samples of phantom B were prepared for synchrotron-CT (sCT) scanning in wet and dry conditions. In each case, samples measured approximately 1 mm diameter × 5 mm length. Wet samples had been immersed in deionised water for 1 week prior to scanning and then inserted into a 20 µl pipette tip, filled with deionised water. The tubing was positioned against the walls of the tip to restrict movement during scanning and the tip was then sealed with Parafilm. Dry samples could be scanned without a pipette tip. Both samples could then be mounted on the magnetic stage for scanning.

Scanning conditions

Tomography was performed at the Diamond-Manchester Imaging Beamline I13-2 of Diamond Light Source, UK. A filtered (950 µm C, 2 mm Al) polychromatic beam (5−35 keV) with parallel geometry was used for imaging (1). Acquisition of phase contrast tomography data was performed using a pco.edge 5.5 detector (PCO AG, Germany) coupled to a CdWO4 scintillator (0.75 mm thick) positioned 45 mm away from the sample; final magnifications for acquisition were ×1.25 and ×10 magnification (FOVs 6.7 mm × 5.6 mm and 0.83 mm × 0.70 mm, respectively). This provided a pixel size of 2.6 µm and 0.33 µm, respectively. The sample was rotated 180° with 0.05° between each exposure (0.1−0.25 seconds). 3600 projection images were acquired for each sample and reconstructed with flat and dark field correction (2).

sCT analysis

The ×10 magnification wet and dry datasets were used to characterise the sphere wall thickness and sphere volume fraction, respectively, due to the differing contrast in images from the two conditions (see Figure 8 in the main text). All analyses were carried out with Fiji (3, 4). Manual measurements of the wall thickness were made on 50 spheres, with the mean of 4 measurements taken as an estimate of the thickness for each sphere. Perpendicular lines through a sphere centre were used to guide the placement of the 4 measurements, which were also guided by overlaying the lines on a 3 × 3 mean filtered image where the boundaries were more distinct; this is illustrated in Figure S1 for an example case. The 50 spheres were chosen by randomly selecting 10 slices throughout the acquired volume, and randomly placing 5 small boxes on each slice; one sphere within each box was then measured, as long as perpendicular lines could be reliably placed such that the four positions on the sphere's outer edge were not obviously merged with other spheres. The final wall thickness estimate was taken as the mean ± SD of the resulting 50 values.

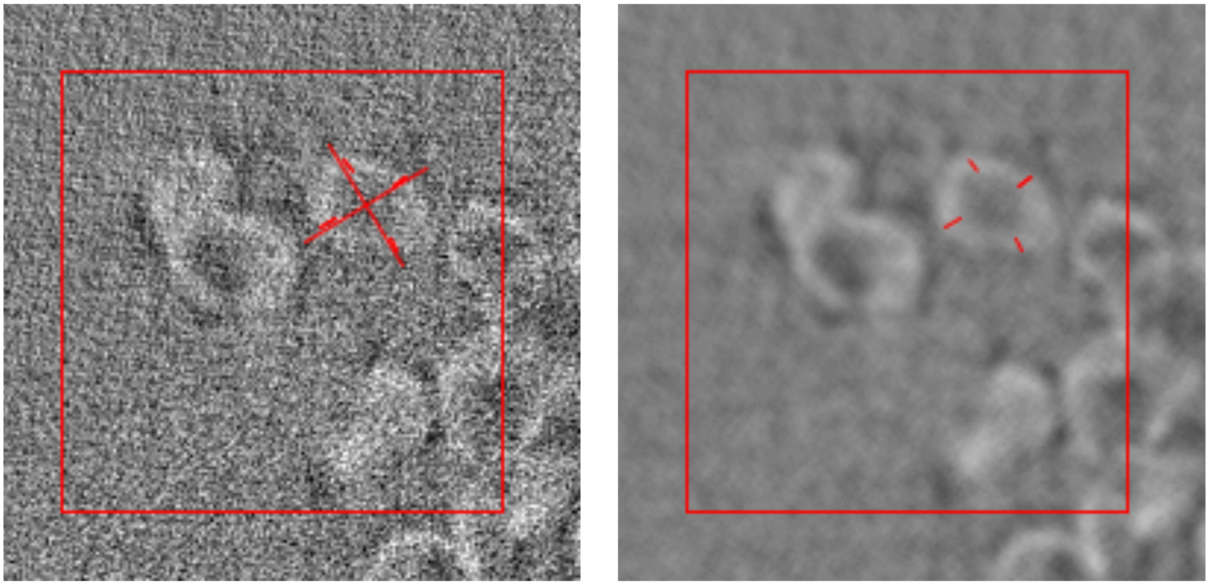

FIG. S1. Wall thickness measurement for phantom B. Four measurements were made for each sphere, with positions guided by perpendicular lines through the sphere centre, as well as by comparing overlays on original (left) and mean filtered (right) images.

The sphere volume fraction was estimated from area fraction measurements made by segmenting the dry-condition images into 'sphere' and 'non-sphere' regions using the Trainable Weka Segmentation plugin in Fiji (5). For this, one slice was picked at random, and sparse manual labelling of 'sphere' and 'non-sphere' regions was performed after cropping excess background (see Figure S2). This labelled image was used to train a classifier, which generated a fully segmented image for the chosen slice. This classifier was then applied to 49 other randomly selected slices, resulting in 50 images segmented into 'sphere' and 'non-sphere' regions. While there was a tendency in some cases to misclassify the central area of spheres as background, due to the low signal inten-

sities in these regions, this could partially be accounted for by applying morphological hole filling to the segmentations. Although some misclassified regions remained, visual assessment of these segmentations indicated that this method consistently produced higher-quality segmentations than alternative methods such as simple thresholding or watershed approaches. On the 50 segmented slices, sphere area fractions, a_f, were obtained from 3 square ROIs, whose in-plane size matched that of an MR voxel (234 µm). The final sphere volume fraction estimate was taken as the mean $\pm$ SD of the resulting 150 a_f values. Note that this reflects both the volume of the sphere wall and the genuine intra-sphere volume, and as such is expected to be larger than the MR-estimated intracellular volume fraction, which is assumed to be insensitive to the sphere wall.

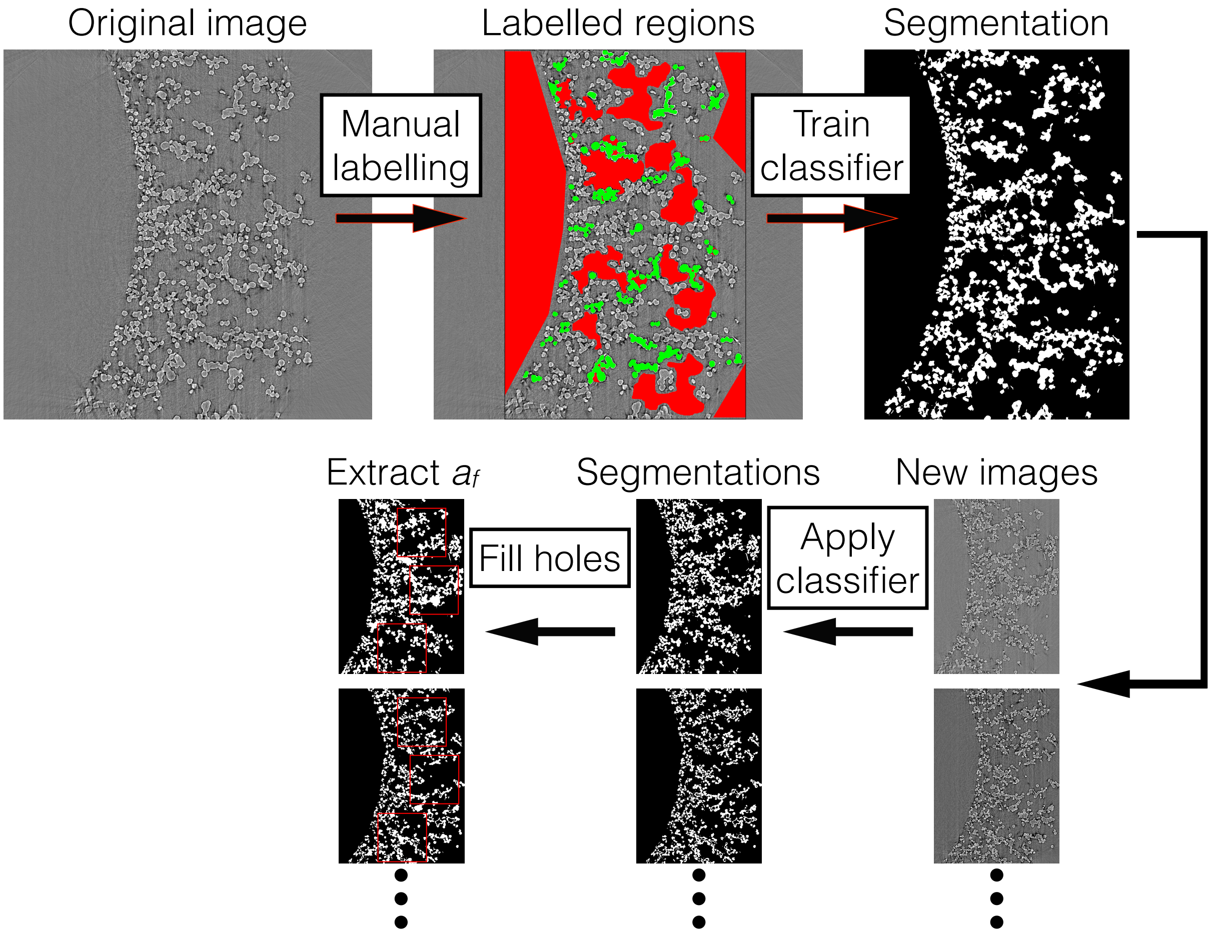

FIG. S2. sCT segmentation for estimating phantom B's sphere volume fraction. On a single cropped slice, manual labelling of 'sphere' (green) and 'non-sphere' (red) regions was performed, and used to train a classifier to segment the entire image. The classifier was then applied to 49 other slices, providing segmentations from which regional area fractions, a_f, were obtained.

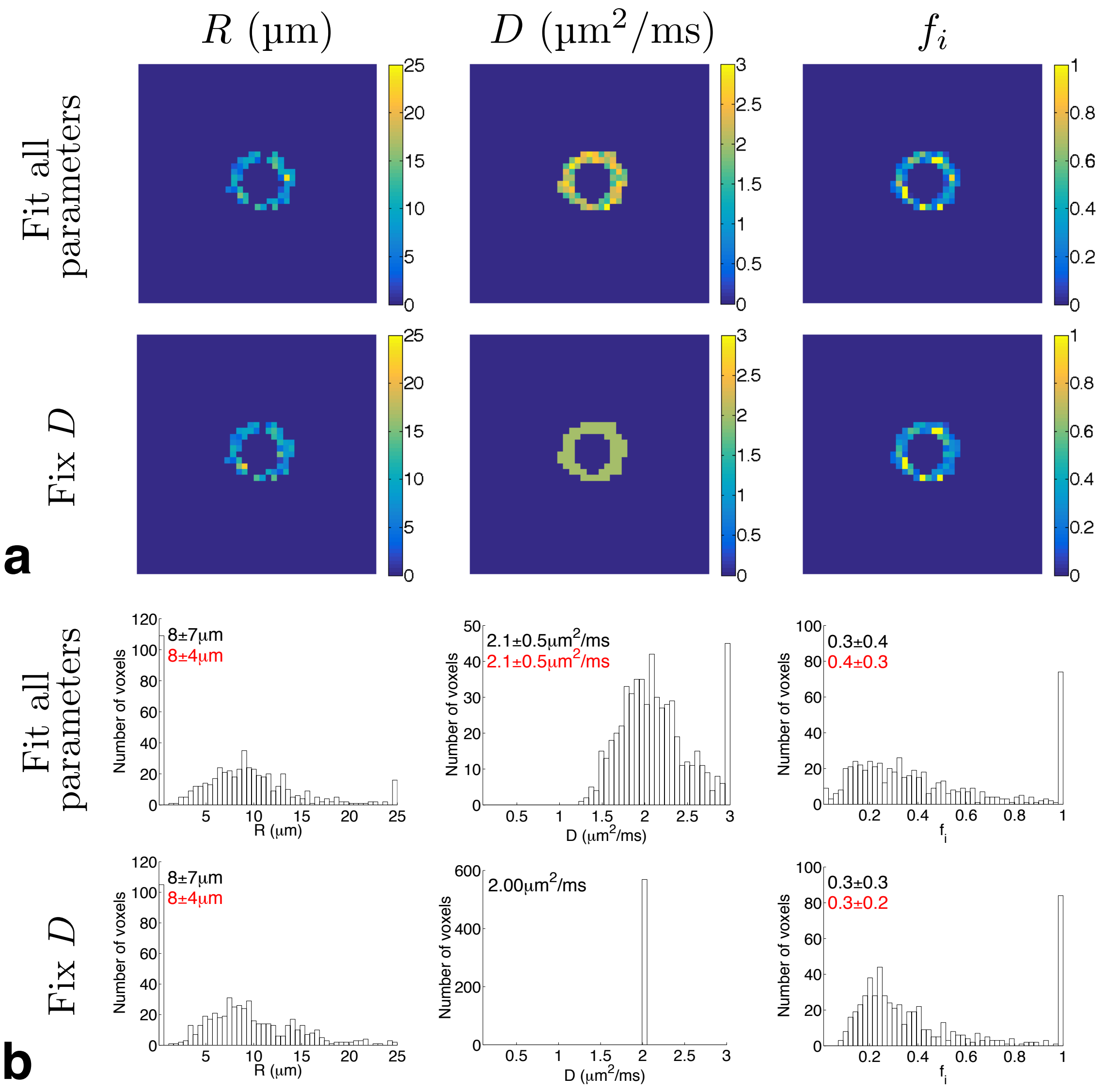

FIG. S3. Voxel-wise microstructural estimates, for phantom A. (a) Example maps of each model parameter are shown (columns) for two fitting procedures (rows). Images are a representative slice from the 1 week time point. (b) Histograms of each model parameter (columns) for two fitting procedures (rows). Quoted values are the median ± IQR, for all phantom voxels (black) and, where applicable, excluding voxels where at least one parameter value was within 1% of the fit constraints (red). The first and third row form Figure 4 in the main text.

References

1. Rau C, Wagner U, Pešić Z, De Fanis A. Coherent imaging at the diamond beamline I13. Phys Status Solidi A 2011;208:2522–2525.

2. Atwood RC, Bodey AJ, Price SWT, Basham M, Drakopoulos M. A high-throughput system for

high-quality tomographic reconstruction of large datasets at Diamond Light Source. Phil Trans R Soc A 2015;373:20140398.

3. Schneider CA, Rasband WS, Eliceiri KW. NIH Image to ImageJ: 25 years of image analysis. Nat Methods 2012;9:671–675.

4. Schindelin J, Arganda-Carreras I, Frise E, et al. Fiji: an open-source platform for biological-image analysis. Nat Methods 2012;9:676–682.

5. Arganda-Carreras I, Kaynig V, Rueden C, Eliceiri KW, Schindelin J, Cardona A, Seung HS. Trainable Weka Segmentation: a machine learning tool for microscopy pixel classification. Bioinformatics 2017. doi: 10.1093/bioinformatics/btx180.
